# Economic Complexity: Correlations between Gross Domestic Product and Fitness

**DOI:** 10.3390/e20100766

**Published:** 2018-10-07

**Authors:** Gianni Valerio Vinci, Roberto Benzi

**Affiliations:** Dipartimento di Fisica, University di Roma “Tor Vergata”, 00133 Roma, Italy

**Keywords:** causal relation, economic growth, economic fitness

## Abstract

In this paper we study the causal relation between country Economic Fitness $F_{c}$ and its Gross Domestic Product *per capita* (GDP). Using the Takens’ theorem, as first suggested in (Sugihara, G. et al. 2012), we show that there exists a reasonable evidence of causal correlation between GDP and $F_{c}$ for relatively rich countries. This is not the case for relatively poor countries where $F_{c}$ and GDP do not show any significant causal relation. We also present some preliminary results to understand whether GDP or $F_{c}$ are driving factor for economic growth.

## 1. Introduction

In their paper, Hidalgo and Hausmann [1] considered the world trade commerce from a new and interesting perspective. In particular they considered the export data from country *c* of product *p* as a bipartite network (worldwide) in which countries are connected to the products they export. Mathematically, the network is represented by the adjacency matrix $M_{cp}$ where $M_{cp}=1$ if country *c* is a significant exporter of product *p* and 0 otherwise. Two basic indicators are then constructed, namely the country diversification $K_{c0}$ and the product ubiquity $K_{p0}$ defined as

(1) $$K_{c0}=\Sigma_{p}M_{cp};\;\;\;\;K_{p0}=\Sigma_{c}M_{cp}.$$

**Figure 1:**
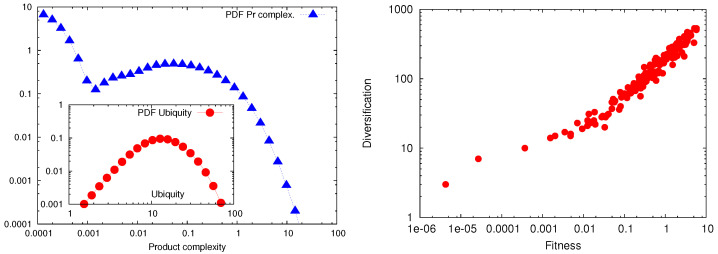
**Left Panel**: Probability distribution of product complexity $Q_{p}$ as obtained from the data set corresponding to period 2000–2012. In the insert we show the probability distribution of product ubiquity $K_{p0}$ defined using Equation (1). Notice that $K_{p0}$ spans over 2 decades and low values of product ubiquity are as rare as large values. This contrasts the probability distribution of $Q_{p}$ which spans over almost 5 decades Furthermore the probability of simple products (i.e., low $Q_{p}$) is much higher than the probability of very complex product (large $Q_{p}$). This implies that the definition of country fitness given by Equation (2) is able to extract more information from the bipartite network $M_{cp}$ with respect to country diversification defined in Equation (1). **Right Panel**: Scatter plots of country fitness (horizontal axis) and country diversification. For relatively large values of $F_{c}$ we observe a scaling $K_{c0}∼F_{c}^{0.65}$ while for low $F_{c}$ (the poverty trap) we observe a different behavior. The scaling law for large fitness suggests that $K_{c0}$ compresses the available informations on country competitiveness with respect to $F_{c}$ as suggested from the results obtained in the left panel.

The analysis performed in [1,2] showed that countries commonly considered as rich and competitive are also characterized by high diversification of their export basket. Furthermore, in the same paper it was argued that the complexity of country economy can be disentangled in term of hidden capabilities which drive the country competitiveness. A different approach was taken by Pietronero and collaborators [3,4,5] where two different quantities, country fitness $F_{c}$ and product complexity $Q_{p}$ are introduced, where the subscripts *c* and *p* refer to the country product names respectively. The country fitness and product complexity can be computed as the fixed points of the non linear iterative maps:

(2) $$\begin{array}{ccc} {\tilde{F}}_{c}^{n+1} & = & \Sigma_{p}M_{cp}Q_{p}^{n} \end{array}$$

(3) $$\begin{array}{ccc} {\tilde{Q}}_{p}^{n+1} & = & \frac{1}{\Sigma_{c}\frac{M_{cp}}{F_{c}^{n}}} \end{array}$$

(4) $$\begin{array}{ccc} F_{c}^{n} & = & \frac{{\tilde{F}}_{c}^{n}}{{\langle {\tilde{F}}_{c}\rangle }_{c}} \end{array}$$

(5) $$\begin{array}{ccc} Q_{p}^{n} & = & \frac{{\tilde{Q}}_{p}^{n}}{{\langle {\tilde{Q}}_{p}\rangle }_{p}} \end{array}$$

where ${\langle \ldots \rangle }_{x}\equiv N_{x}^{-1}\Sigma_{x}\ldots$ and $N_{x}$ is the number of countries in (4) and products in (5), see [3] for details. It is quite easy to show that Equations (2) and (3) are self consistent, i.e., once we assume (2) then Equation (3) follows from the constrain on the finite number of countries and products. At variance with Equation (1) where country diversification and product ubiquity are defined independently, country fitness and product complexity are linked together in a non linear way, suggesting that we may be able to extract more informations from the bipartite network. This suggestion seems correct as demonstrated in the two panel of Figure 1.

In the left panel of Figure 1, we show the probability distribution of product complexity $Q_{p}$ obtained using (2)–(5) and collecting the results on about 20 years. In the insert of the same panel we plot the probability distribution of product ubiquity $K_{p0}$ obtained for the same data set (i.e., the matrices $M_{cp}$ for the same period of time). The figure shows two different interesting features: (1) the values of $Q_{p}$ span on about 5 decades while the variation of $K_{p0}$ is limited by the two decades corresponding to the number of countries; (2) product with large ubiquity $K_{p0}$ are as rare as product with small ubiquity while very complex products measured by $Q_{p}$ are extremely rare with respect to low complex products. These two observations implies that country fitness can disentangle small variations in country competitiveness at variance with the indicator $K_{c0}$ limited by the number of products. In the right panel of Figure 1, we support the above conclusion by showing the scatter plot of $K_{c0}$ versus $F_{c}$. A clear scaling relation is observed for large $F_{c}$ in the form $K_{c0}∼F_{c}^{0.65}$. The scaling is however changed for small value of $F_{c}$ (to be identified with the so called poverty trap [6]) where the slope is much smaller. The results shown in Figure 1 definitively suggests that, at least in principle, more information is gained using $F_{c}$ and $Q_{p}$ with respect to the original proposal discussed in [1].

**Figure 2:**
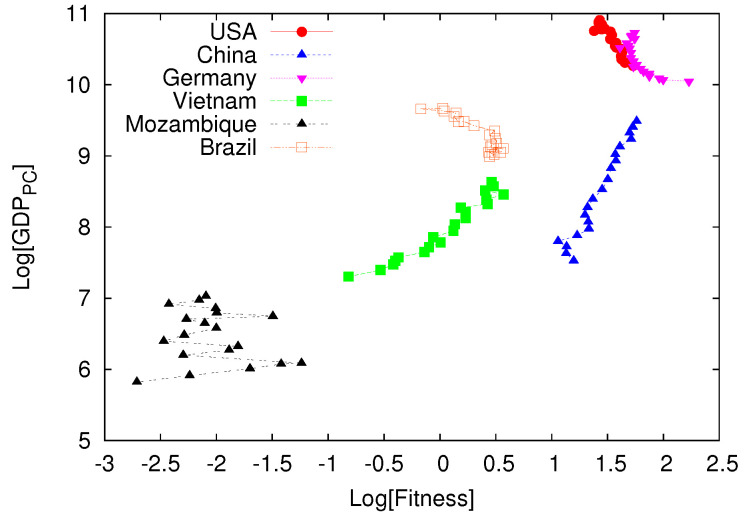
Trajectories of GDP as a function of country fitness $F_{c}$ from a selected number of countries. While relatively rich countries (right upper corner) show small fluctuations in both GDP and $F_{c}$, fast growing economies, like China and Vietnam, show a well defined *laminar* flow towards the upper right corner. Other countries, like Brazil, stops increasing their fitness and their GDP in the last few years. Finally, poor countries, like Mozambique, shows a rather erratic or chaotic behavior for both variables.

One important point, which is the basic subject of this paper, is whether the information contained in $F_{c}$ and $Q_{p}$ can be eventually related to the most used indicator of country competitiveness given by the Gross Domestic Product GDP. Hereafter we consider the GDP per capite. A first answer to this question is shown in Figure 2 following the original proposal in [4]. In Figure 2, we plot for a selected number of countries the trajectories in the place $F_{c},GDP$ obtained from a time series of about 20 years. While rich countries, like USA and Germany, perform small oscillations in the upper right corner of the figure, the interesting features emerges looking at China and Vietnam which are moving in a regular way towards the “rich” countries region. The same is not true for Brazil which definitively stops its country competitiveness (measured by $F_{c}$) and decreasing its GDP. In bottom left corner (the “poor” corner) we plot the results obtained from Mozambique which shows a rather chaotic or irregular behavior.

The above discussion opens the question whether there is or not a causal relationship between $F_{c}$ and GDP and whether we can eventually predict one from the other. The interpretation of Figure 2 suggests that this is indeed the case. In this paper, following the proposal in [5], we show that is is possible to measure the causal relation between $F_{c}$ and GDP, using the tools introduced in [7,8]. Since our investigations are constrained within the present limitation of available data, we consider our results as first but non trivial and positive result.

## 2. Investigating Causality in $F_{c}$-GDP Relation

To compute causal relationship between $F_{c}$ and GDP we follow the method described in [7]. The basic idea of the method is rather intuitive. Let us consider a system described by 2 variables say *X* and *Y* and let us assume that in the plane X,Y the system performs some generic chaotic motion. There is no need a priori to assume that the motion is chaotic or, at least, that chaos is induced by the linear or non-linear coupling between X,Y (there is in principle no limitation on the number of variables). Using Takens theorem we know that it is possible to reconstruct the attractor in the phase space X,Y by looking at the behavior of *X* or *Y* independently. In practice, we assume to have a time series $X_{i}$ and we consider the vectors $V_{X}\left(i\right)=\left[X_{i},X_{i+1},\ldots X_{i+E}\right]$ where *E* is the embedded dimension. For *E* greater than attractor dimension (which may be a fractal dimension) and assuming that the system can be described with a diffeomorphism, we can reconstruct the original attractor by the vectors $V_{X}\left(i\right)$. Let us now suppose to pick up one of this vector, say $V_{X}\left(n\right)$ and to look at the corresponding vector $V_{Y}\left(n\right)$. Because of Takens theorem, we can state that the values of $V_{Y}\left(n\right)$ can be reconstructed accurately by using those of $V_{X}\left(n\right)$. Let us call $V_{Y}^{S}\left(n\right)$ the values of $V_{Y}\left(n\right)$ by using $V_{X}\left(n\right)$. Then the correlation $\rho_{XY}$ between $V_{Y}\left(n\right)$ and $V_{Y}^{S}\left(n\right)$ is a quantitative measure of the causal relationship between *X* and *Y*. The same can be done to reconstruct *X* from *Y* measured by $\rho_{YX}$. If *X* drives *Y* than $\rho_{YX}>0$. This method was successfully verified in simple chaotic systems and later applied to explain rather non trivial observations in marine ecology. In the rest of the paper we follow the same notation of [7] i.e., $X\left(t\right)|M_{Y}$ reads *X* reconstructed from *Y* or, as is often said, *X* cross map *Y*.

When applying the method proposed in [7] in our case, we are faced with two different problems: one problem is data availability which is a sever limitation even with respect to the above mentioned ecological applications; the second problem concerns the choice of the embedding dimension *E*. In fact the two problems are connected: if we assume that our data set gives just a partial informations on the relevant degrees of freedom, then we need to increase the embedding dimension *E* up to the value where the quantitative computations are independent of *E*. This implies a longer data set. In our case, as well in other possible applications, data set is the major constrain. Thus, we are forced to assume that $F_{c}$ and GFP are the most relevant variables in describing economic competitiveness at least on the world wide trade. It follows that in our case E=2. Whether this assumption is reasonably fair or not can be judged by the results.

**Figure 3:**
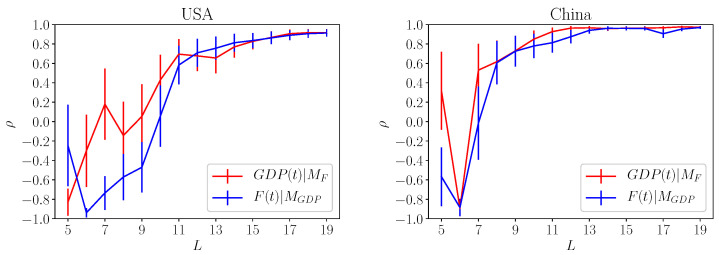
Left and right panels refers to the causal correlation obtained for Usa (**left**) and China (**right**). The quantity $GDP\left(t\right)|M_{F}$ indicates the correlation $\rho_{F,GDP}$ obtained by reconstructing GDP from the country fitness $F_{c}$. The quantity $F\left(t\right)|M_{GDP}$ refers to the correlation of $F_{c}$ with the values obtained by reconstructing country fitness using GDP. For both countries we observe a rather large correlations indicating the there is a clear causal connection between the two variables.

**Figure 4:**
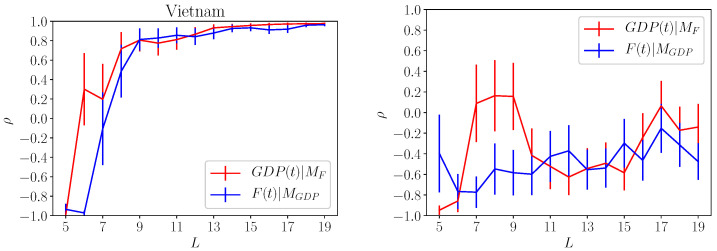
Same results of Figure 3 obtained for Vietnam (**left panel**) and Mozambique (**right panel**). While Vietnam shows large values of the correlation for both $GDP\left(t\right)|M_{F}$ and $F\left(t\right)|M_{GDP}$ the same is not true for Mozambique. This result suggests that the GDP and $F_{c}$ alone may not be able to explain country economy. Also, upon comparing Mozambique result against China, Usa and Vietnam, we can reasonably argue that our results are significant despite the limited data set used in the analysis.

We have applied the method proposed in [7] for a selected set of countries and the results are shown in Figure 3 and Figure 4. In Figure 3, we consider the quantity $\rho_{F,GDP}$ (red lines) and $\rho_{GDP,F}$ (blue line) for USA and China. The error bars for the each cases, shown as vertical line, decrease by increasing the size of the data set. Although for the whole data set the error bars are approximately of order 15%, it is quite clear that we observe a rather strong causal effect both ways.

In Figure 4, we show the results for Vietnam and Mozambique (right side). While the causal effect *F*-GDP is rather strong for Vietnam, the same is not true for Mozambique which clearly shows a very poor correlation both ways. The last case is interesting since it proves that the correlations displayed in Figure 3 and for the Vietnam is significant even with the large error bars. Also the poor results obtained for the Mozambique suggests that the embedding dimension can be larger than 2. This is equivalent to say that other variables, not included in our computation of *F* and GDP, should be considered to analyze the Mozambique economy. Let us remark that for the Mozambique case, the error bars are not decreasing by increasing the data set.

**Figure 5:**
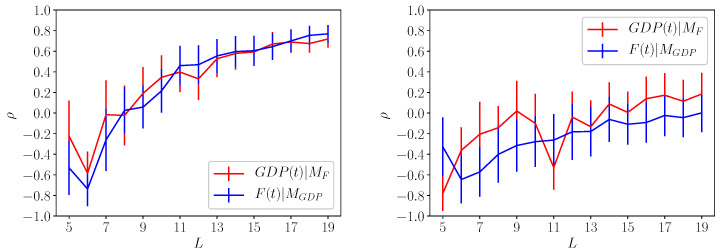
**Left Panel**: Causal correlations $GDP\left(t\right)|M_{F}$ and $F\left(t\right)|M_{GDP}$ obtained as the average over 10 relatively rich countries of individual correlations. **Right Panel**: Same as the left panel obtained upon averaging correlations from countries in the poverty trap. The results shown on the left panel are consistent with the ones obtained for individual countries displayed in Figure 3 and Figure 4 (left panel): a rather large correlation is observed in all cases. Because of averaging, the error bars is now reduced to values order 10%. This implies that GDP and country fitness $F_{c}$ can be considered in causal correlation. The results shown on the right panel of the figure suggests that for countries in the poverty trap and corresponding to the *chaotic* behavior observed on the left bottom corner of Figure 2, GDP and country competitiveness $F_{c}$ may not be the only variables to consider for a proper description.

We can improve our result by averaging the quantity $\rho_{F,GDP}$ and $\rho_{GDP,F}$ over an homogeneous set of countries. In this way we can reasonably reduce the overall error bars in our estimation. In particular we consider about 10 countries in the so called right “rich” corner of Figure 2 (including China and Vietnam) and a similar number in the right “poor” corner. Upon averaging the correlations on the number of countries, we obtain the results displayed in Figure 5. We can now estimate the error bars of the order of 10%. This figure represents the most relevant result obtained in this paper: for relatively “rich” countries there is quite significant causal correlation between country fitness and GDP while this is not the case for “poor” or underdevelopment countries. We consider Figure 5 an important result because it clearly supports most of the suggestions and the analysis performed in [3,4] to assess the quantity $F_{c}$ as a robust measure of county competitiveness. It also suggests that the case involving poor or underdevelopment countries should be analyzed case by case without any obvious generalization from one case to the other. For all the studies in this section the cross mapping was done considering no time lag in the time series of the two variables.

## 3. Effects on Different Time Lags

Once we have showed the causal relationship *F-GDP*, we look at whether we can predict one from the other. This is a rather difficult question to investigate although it is a crucial question for any possible applications of the method proposed in [3]. Takens’theorem suggests that, in principle, it should be possible to reconstruct the manifold of a given variable starting from another, belonging to the same dynamical system, even if the former is considered with some time lag (i.e., Y(t) and X(t+m)). Following this idea, it was proposed in [8] an extended version of the algorithm discussed in [7] using a lagged time series in order to guess the optimal time lag *m* among different variables. If some sort of causality between two variables holds, it is natural to expect the optimal time lag to be negative. So if for example *X* causes *Y*, most of the information on Y(t) at present time must be related to X(t−m). Therefore we would have an higher value of $\rho_{XY}$ for the reconstruction of X(t−m) using Y(t). Note that the logic may be confusing but it is straightforward: if the variations of *Y* are due to *X* then it should be easy to guess the value of *X* knowing *Y*. This is the reason to reconstruct X(t−m) using Y(t).

Here we present some preliminary results obtained by applying the above mentioned idea in our case. We consider to different possibilities: (1) country fitness at time t−m (expressed in years) and GDP at time *t* and (2) GDP at time t−m and country fitness *F* at time *t*. For the first case we are interested to the correlation $\rho_{GDP,F}$ which, in the following we denote by $F\left(t\right)|M_{GDP}$. In the second case we compute $\rho_{F,GDP}$ which we denote by $GDP\left(t\right)|M_{F}$. Last, in the following figure we denote by lag the quantity −m, i.e., negative lag corresponds to the past and positive lag to the future. In summary:

- when $F\left(t\right)|M_{GDP}$ shows a significant peak for *negative* lag, then we may argue that country fitness *F* at time t+lag has a causal effect on GDP at time *t*; consequently we must find a significant peak $GDP\left(t\right)|M_{F}$ for *positive* lag;
- when $GDP\left(t\right)|M_{F}$ shows a significant peak for *negative* lag, then we may argue that country fitness GDP at time t+lag has a causal effect on GDP at time *t* and, as in the previous case, we must find a significant peak $F\left(t\right)|M_{GDP}$ for *positive* lag.

Needless to say, for countries similar to Mozambique we do not expect any significant peak.

**Figure 6:**
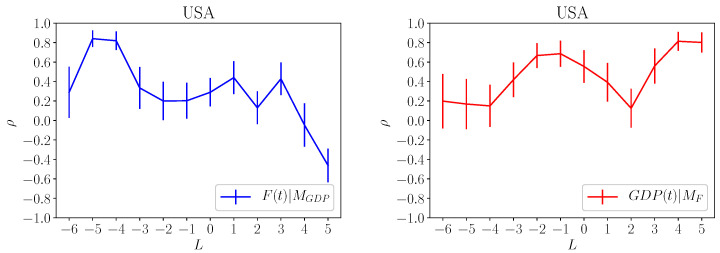
**Left Panel**: Correlation $F\left(t\right)|M_{GDP}$ obtained by reconstructing *F* at time t+lag using GDP at time *t*. As described in the text, large correlation for *negative*lag corresponds to a causal connection of $F_{c}\left(t+lag\right)$ and GDP(t), i.e., country fitness is the cause of GDP. **Right Panel**: Same as the left panel for the correlation $GDP\left(t\right)|M_{F}$ where $F_{c}$ is computed at time *t* and GDP at time t+lag. Data correspond to USA economy.

In Figure 6, we show the results obtained for the USA data set: on the left panel we show $F\left(t\right)|M_{GDP}$ while on the right panel $GDP\left(t\right)|M_{F}$. On the left panel, there is a clear peak at negative lag for the correlation $F\left(t\right)|M_{GDP}$ which means that an increase of country fitness causes an increase of GDP on a time scale of 4 years. On the right panel we observe a peak for negative lag for the correlation $GDP\left(t\right)|M_{F}$ at time lag of 2 years and a secondary peak at positive lag at year 4. The first peak corresponds to the causal effect of the GDP on fitness and, interesting, it occurs on a shorter time lag with respect to the one observed on the left panel. The second peak on the right panel has the same meaning of the peak observed on the left panel, i.e., it corresponds on the causal effect of fitness on GDP for a time scale close to 4 years. Summarizing the above observations, we can tentatively conclude that in the USA economy fitness and the GDP are tightly connected in a virtuous circle: on relative long time scale (about 4 years) an increase in the fitness will increase the GDP. On shorter time scale GDP foster the country fitness.

The results discussed in this section are rather speculative. On the one hand, we can confirm the existence of clear causal correlation between country fitness and GDP already investigated in the previous section. On the other hand, we can have a reasonable hint on the way GDP and fitness can mutually interact on different time scales within the single country. Again, as previously discussed, data availability constrains our findings and we must accept large error bars in our quantitative results. Nevertheless it is worth noting that one can detect non trivial differences among different countries. These differences can be investigated in a deeper way upon considering other possible informations able to provide an in depth understanding of country economy.

**Figure 7:**
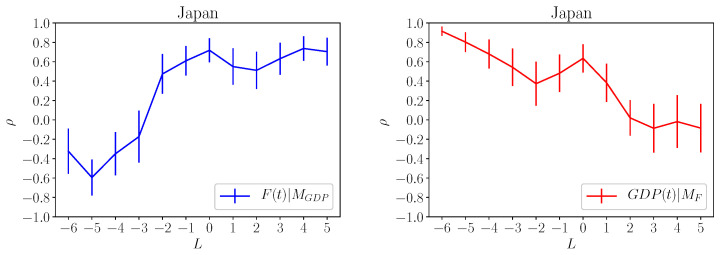
Same as in Figure 6 for the Japan economy.

The same is not true for other countries. In Figure 7, we show the results obtained for Japan. From both panel we can reach the same conclusion, namely that the GDP is driving the country fitness on a time scale of 5 years. The reason for the differences found between USA and Japan economy deserve a deeper analysis which is, however, outside the aim of this paper.

## 4. Discussion

In this paper we have analyzed the causal correlation between GDP (gross domestic product *per capita* and country competitiveness measured in terms of country fitness $F_{c}$, see Equation (2)). Although the data set is rather limited, we have been able to show that there exists a clear causal connection between GDP and $F_{c}$. The most important result is shown in Figure 5. The causal connection is obtained employing the method discussed in [7] and assuming that GDP and $F_{c}$ are the only variables needed to describe the overall country performance. The high correlations shown in Figure 5 (left panel) suggest that our assumption is correct and that GDP and $F_{c}$ are definitively causal correlated for relatively rich countries. This is not the case for relatively poor countries where other economic informations are needed to provide a more significant picture.

Causal relation between GDP and $F_{c}$ is a rather important feature. In [4], upon inspecting country trajectories similar to the ones displayed in Figure 2, it was argued that a causal relation GDP and $F_{c}$ should exist and it can be used for a deeper investigation of country performance. Notice that country fitness $F_{c}$ is a global measure of country competitiveness in terms of significant production of complex products. Therefore, following [1,3], $F_{c}$ is an indirect measure of country *hidden* capabilities not directly taken into account by the GDP value.

We also presented some preliminary results on the time lag effect in the causal correlation. For the USA economy we observed that country fitness is correlated to GDP on time scale of about 4 years, i.e., the value of $F_{c}$ at time t−4 (in years) is “predicting” the value of GDP. The same is true for the GDP at time t−2, i.e., an increase or decrease of GDP is directly connected to the present country fitness. We argued that both variables GDP and $F_{c}$ are related with a tight and virtuous circle on different time scales. Clearly, a deeper economic investigation is needed to understand whether our claim can be considered correct and, eventually, how it can be used. For other countries, like Japan, the GDP is the variable determining the country competitiveness $F_{c}$ within a time scale of 5 years. Similar results, not shown, are true for other “rich” countries like France or Germany. The difference between USA economy and Japan economy (representative of a large number of countries) opens an interesting question to be investigated in the future.

## Abbreviations

The following abbreviations are used in this manuscript:

MDPI	Multidisciplinary Digital Publishing Institute
DOAJ	Directory of open access journals
TLA	Three letter acronym
LD	linear dichroism
